# Effect of Arthroplasty vs Fusion for Patients With Cervical Radiculopathy: A Randomized Clinical Trial

**DOI:** 10.1001/jamanetworkopen.2021.19606

**Published:** 2021-08-05

**Authors:** Tonje Okkenhaug Johansen, Jarle Sundseth, Oddrun Anita Fredriksli, Hege Andresen, John-Anker Zwart, Frode Kolstad, Are Hugo Pripp, Sasha Gulati, Øystein Petter Nygaard

**Affiliations:** 1 Department of Neurosurgery, St Olavs Hospital, Trondheim University Hospital, Trondheim, Norway; 2 Department of Neuromedicine and Movement Science, Norwegian University of Science and Technology, Trondheim, Norway; 3 Department of Neurosurgery, Oslo University Hospital Rikshospitalet, Oslo, Norway; 4 National Advisory Unit on Spinal Surgery, St Olav’s Hospital, Trondheim University Hospital, Trondheim, Norway; 5 Faculty of Medicine, University of Oslo, Oslo, Norway; 6 Division of Clinical Neuroscience, Department of Research and Innovation, Oslo University Hospital, Oslo, Norway; 7 Research Support Services, Oslo Centre of Biostatistics and Epidemiology, Oslo University Hospital, Oslo, Norway

## Abstract

**Importance:**

Surgical treatment for cervical radiculopathy is increasing. Treatment with motion preserving anterior cervical disc arthroplasty was introduced to prevent symptomatic adjacent segment disease, and there is need to evaluate results of this treatment compared with standard anterior cervical discectomy and fusion.

**Objective:**

To investigate clinical outcomes at 5 years for arthroplasty vs fusion in patients who underwent surgical treatment for cervical radiculopathy.

**Design, Setting, and Participants:**

This multicenter, single-blinded, randomized clinical trial included patients aged 25 to 60 years with C6 or C7 radiculopathy referred to study sites’ outpatient clinics from 2008 to 2013. Data were analyzed from December 2019 to December 2020.

**Interventions:**

Patients were randomly assigned to arthroplasty or fusion. Patients were blinded to which treatment they received. The surgical team was blinded until nerve root decompression was completed.

**Main Outcomes and Measures:**

The primary end point was change in Neck Disability Index (NDI) score. Secondary outcomes were arm and neck pain, measured with numeric rating scales (NRS); quality of life, measured with the EuroQol-5D (EQ-5D); reoperation rates; and adjacent segment disease.

**Results:**

Among 147 eligible patients, 4 (2.7%) declined to participate and 7 (4.8%) were excluded. A total of 136 patients were randomized (mean [SD] age, 44.1 [7.0] years; 73 (53.7%) women), with 68 patients randomized to arthroplasty and 68 patients randomized to fusion. A total of 114 patients (83.8%) completed the 5-year follow-up. In the arthroplasty group, the mean NDI score was 45.9 (95% CI, 43.3 to 48.4) points at baseline and 22.2 (95% CI, 18.0 to 26.3) points at 5 years follow-up, and in the fusion group, mean NDI score was 51.3 (95% CI, 48.1 to 54.4) points at baseline, and 21.3 (95% CI, 17.0 to 25.6) points at 5 years follow-up. The changes in mean NDI scores between baseline and 5 years were statistically significant for arthroplasty (mean change, 24.8 [95% CI, 19.8 to 29.9] points; *P* < .001) and fusion (mean change, 29.9 [95% CI, 24.0 to 35.9] points; *P* < .001), but the change in mean NDI scores was not significantly different between groups (difference, 5.1 [95% CI, −2.6 to 12.7] points; *P* = .19). There were no significant differences in changes in arm pain (mean [SE] change, 3.5 [0.5] vs 3.1 [0.4]; *P* = .47), neck pain (mean [SE] change, 3.0 [0.5] vs 3.4 [0.5]; *P* = .50), EQ-5D (mean [SE] change, 0.39 [0.4] vs 0.45 [0.6]; *P* = .46), patients requiring reoperation (10 patients [14.7%] vs 8 patients [11.8%]; *P* = .61), and adjacent segment disease (0 patients vs 1 patient [1.5%]; *P* = .32) between the arthroplasty and fusion groups.

**Conclusions and Relevance:**

In this randomized clinical trial, patients treated with arthroplasty and fusion reported similar and substantial clinical improvement at 5 years.

**Trial Registration:**

ClinicalTrials.gov Identifier: NCT00735176

## Key Points

**Question:**

In patients with cervical radiculopathy, are there any differences in long-term outcomes after surgery with arthroplasty or fusion?

**Findings:**

In this randomized clinical trial that included 136 patients with cervical radiculopathy, there were similar and significant improvements in Neck Disability Index scores for both treatment groups at the 5-year follow-up. There was no statistically significant difference in change of Neck Disability Index score at 5 years between patients who underwent arthroplasty and fusion.

**Meaning:**

These findings suggest that arthroplasty and fusion are both good treatment options for cervical radiculopathy and have similar long-term efficacy.

## Teaser

This randomized clinical trial examines 5-year outcomes among patients who underwent surgical treatment for cervical radiculopathy with arthroplasty vs fusion.

## Introduction

Cervical radiculopathy is a frequently encountered neurologic condition and is most often caused by encroachment of a cervical nerve root. It typically presents with neck and arm pain and sometimes sensory loss or loss of motor function in the affected nerve root distribution.^1^ The most common cause of cervical radiculopathy is degenerative features, including reduced disc height, osteophyte formation, and disc herniation.^2^

Anterior cervical discectomy and fusion either as stand-alone implant surgical treatment or with the use of additional plating has for decades been the criterion standard treatment for cervical radiculopathy,^3^ and more than 100 000 patients receive this treatment in the US annually.^4^ Surgical treatment for cervical radiculopathy is increasing in most countries and is projected to increase by more than 10% in the next 20 years.^5^

In recent years, arthroplasty (ie, artificial disc replacement) designed to maintain normal motion has gained use owing to the concern that fusion may cause adjacent segment disease.^6,7^ Many studies and meta-analyses have compared these 2 treatment options. Most authors presented results in favor of arthroplasty. However, many of the studies were industry sponsored, and few studies blinded patients and/or clinicians.^8,9,10,11^ The Norwegian Cervical Arthroplasty Trial (NORCAT)^12^ blinded both patients and clinicians and found equal and beneficial outcomes for arthroplasty and fusion at 2 years. The aim of this study was to present the 5-year outcomes and reoperation rates in patients who underwent surgical treatment for single-segment cervical radiculopathy with either arthroplasty or fusion.

## Methods

This randomized clinical trial was approved by the Regional Committee for Medical Research and Health Research Ethics in Central Norway, and all participants provided written informed consent. The trial protocol is available in Supplement 1. This study is reported following the Consolidated Standards of Reporting Trials (CONSORT) reporting guideline.

### Trial Design

NORCAT was a single-blinded, multicenter, randomized clinical trial and has previously been described in detail.^12^ In total, 136 patients with C6 or C7 radiculopathy at 5 neurosurgical departments in Norway were randomized to either arthroplasty or fusion using a stand-alone cage after being screened for study eligibility (eTable in Supplement 2). Randomization and data collection were performed by a web-based randomization and data collection system developed and administered by the faculty of Medicine and Health Sciences, Norwegian University of Science and Technology, Trondheim, Norway.^27^ The patients were included in the period between 2008 and 2013. The Discover prosthesis, which allows for unconstrained movement, was used in the arthroplasty group, and the Cervios cage (DePuy Synthes Spine) was used as stand-alone implant in the fusion group. To keep the patients and surgical team blinded, patients were randomized to either arthroplasty or fusion in the operating room after nerve root decompression was completed and the end plate was prepared for implantation of either cage or arthroplasty. The patients were blinded to type of implant and offered an opportunity to know which treatment they received at the 2- and 5-year follow-ups.

### Data Collection

Study participants had follow-up visits scheduled at 3 months, 1 year, and 2 years. Additionally, they answered questionnaires by mail at 6 months and 5 years. Participants answered the questionnaires without supervision or help from any members of the study group.

### Primary Outcome

The primary outcome was change in Neck Disability Index (NDI), a self-rated questionnaire developed for patients with neck disability^13^ that has been translated into Norwegian and tested for psychometric properties.^14^ It comprises 10 items: 7 related to activities of daily living (personal care, lifting, reading, work and daily activities, driving, sleep, and recreation), 2 related to pain (pain and headache), and 1 related to concentration. Each item is rated on a scale from 0 to 5 points. The NDI summary score is typically presented as a score that ranges from 0 to 100, with lower scores indicating less disability.

### Secondary Outcomes

Secondary outcomes included health-related quality of life, neck pain, arm pain, reoperation rate, and adjacent segment disease. Health-related quality of life was measured with the Euro-QoL-5D 3L (EQ-5D).^15^ The Norwegian version of EQ-5D has shown good psychometric properties.^16^ It evaluates 5 dimensions: mobility, self-care, activities of daily living, pain, anxiety, and/or depression on 3 levels (none, mild to moderate, and severe). An index value for health status was generated for each patient. Scores ranged from −0.6 to 1, where 1 indicates perfect health. Neck and arm pain were measured with numeric rating scales (NRS),^17^ a 1-dimensional pain scale from 0, indicating no pain, to 10, worst imaginable pain. Reoperation rates and reasons for reoperations, including adjacent segment, index segment, and other segments, were recorded.

### Statistical Analysis

Statistical analyses were performed with SPSS statistical software version 25 (IBM) For statistical comparison tests, we defined the significance level as *P* = .05 with no adjustments for multiple comparisons. The trial was planned to have 80% power to detect a difference of 10 points in the NDI score, considered to be the minimal clinically important difference, between the 2 groups. Based on a significance level of .05 and an SD of 18, 104 participants were required for the trial. After adjusting for 40% expected loss to follow-up, a total of 146 participants were required.

Patient-reported outcome measures (PROMs) were analyzed according to the intention-to-treat principle. We used paired sample *t* test to look for statistical differences between the groups as equal variance was assumed. Missing data were handled with mixed linear models. This strategy was in line with a study by Twisk et al^18^ showing that multiple imputations are not necessary before performing a mixed-model analysis on longitudinal data and is well established for PROMs in spine surgery.^19,20^ The model adjusted for baseline differences by random intercepts through the course of follow-up. Treatment modalities, baseline scores, and follow-up time points were included as fixed main effects together with interaction terms between follow-up time points and treatment modality. We estimated the mean difference between treatment modalities with 95% CIs at each follow-up time point using linear combinations of estimators. Another reason for using the linear mixed model analysis was differences in NDI scores at inclusion. Data were analyzed from December 2019 to December 2020.

## Results

### Patient Characteristics

**Figure 1.**
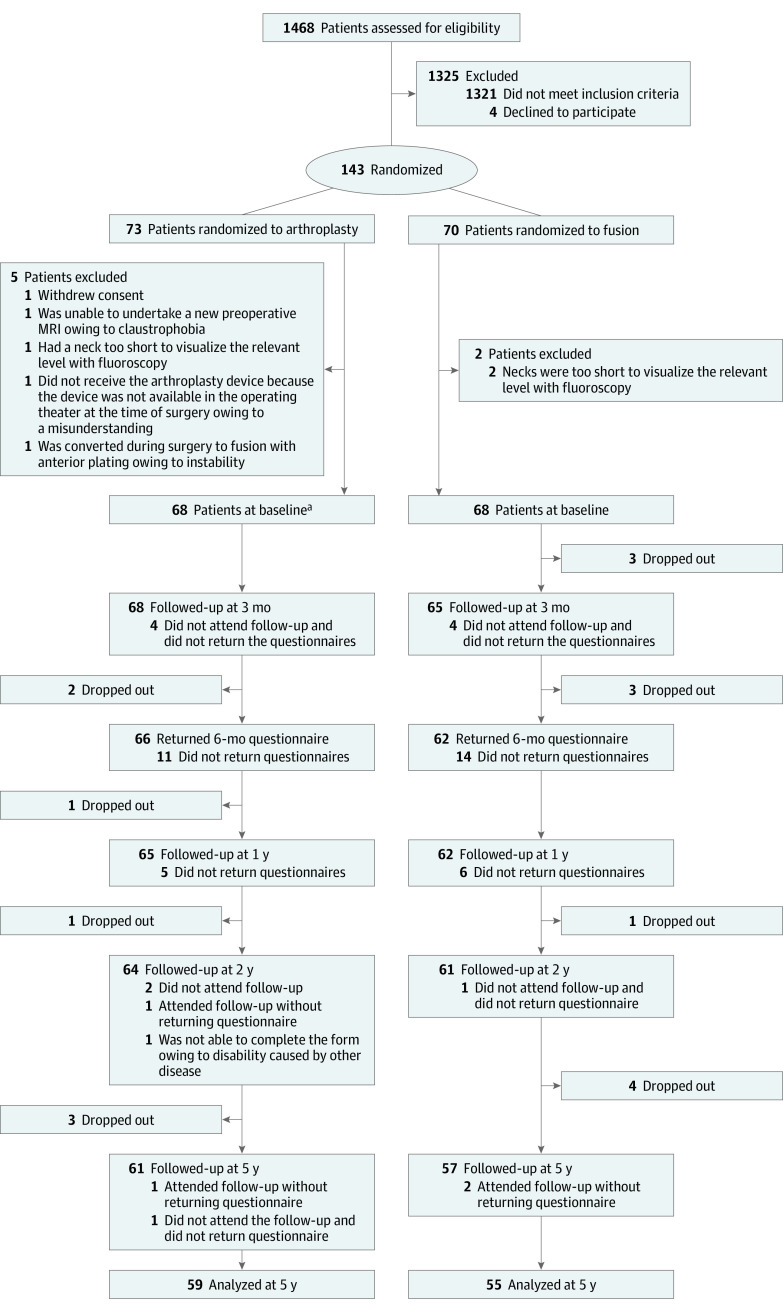
Patient Recruitment Flowchart

Among 147 eligible patients, 4 (2.7%) declined to participate and 7 (4.8%) were excluded. A total of 136 patients with single-segment cervical radiculopathy were included (mean [SD] age 44.1 [7.0] years; 73 [53.7%] women), with 68 patients randomized to arthroplasty and 68 patients randomized to fusion (Figure 1). Baseline characteristics were comparable in the 2 treatment groups, except for fewer smokers in the arthroplasty group compared with the fusion group (23 smokers [34.9%] vs 29 smokers [47.5%]) (Table 1). In total, 114 patients (83.8%) attended the 5-year follow-up, including 59 patients (86.7%) from the arthroplasty group and 55 patients (80.9%) from the fusion group.

**Table 1.** Baseline Characteristics of Included Patients

Characteristic	No. (%)	
	Arthroplasty (n = 68)	Fusion (n = 68)
Age, mean (SD), y	44.7 (7.2)	43.4 (6.8)
Sex		
Women	36 (52.9)	37 (54.4)
Men	32 (47.1)	31 (45.6)
Height, mean (SD), cm	174.1 (10.6)	172.7 (8.9)
Weight, mean (SD), kg	79.1 (14.6)	76.8 (15.8)
BMI, mean (SD)	26.0 (3.7)	25.5 (3.6)
Operated segment C5/C6	38 (55.9)	36 (52.9)
Duration of absence from work, median (range), wk	21 (6-39)	24 (1-55)
Duration of arm pain		
<3 mo	3 (4.5)	6 (9.1)
3 mo-1 y	35 (53.0)	30 (45.1)
1-2 y	14 (21.2)	20 (30.3)
>2 y	14 (21.2)	10 (15.1)
Duration of neck pain		
No neck pain	3 (4.5)	2 (3.0)
<3 mo	4 (6.1)	3 (4.5)
3 mo-1 y	27 (40.9)	28 (41.8)
1-2 y	11 (16.7)	19 (28.4)
>2 y	21 (31.8)	15 (22.4)
Working until surgery	14 (20.6)	17 (25.0)
≥College education	28 (41.2)	26 (38.2)
Smoking, No./No. (%)	23/66 (34.9)	29/61 (47.5)
Married or cohabitating	59 (86.8)	50 (73.5)
Comorbidity		
Heart disease	1 (1.5)	1 (1.5)
Hypertension	6 (8.8)	2 (2.9)
Diabetes	4 (6.1)	3 (4.4)

Abbreviation: BMI, body mass index (calculated as weight in kilograms divided by height in meters squared).

### Primary Outcome

**Figure 2.**
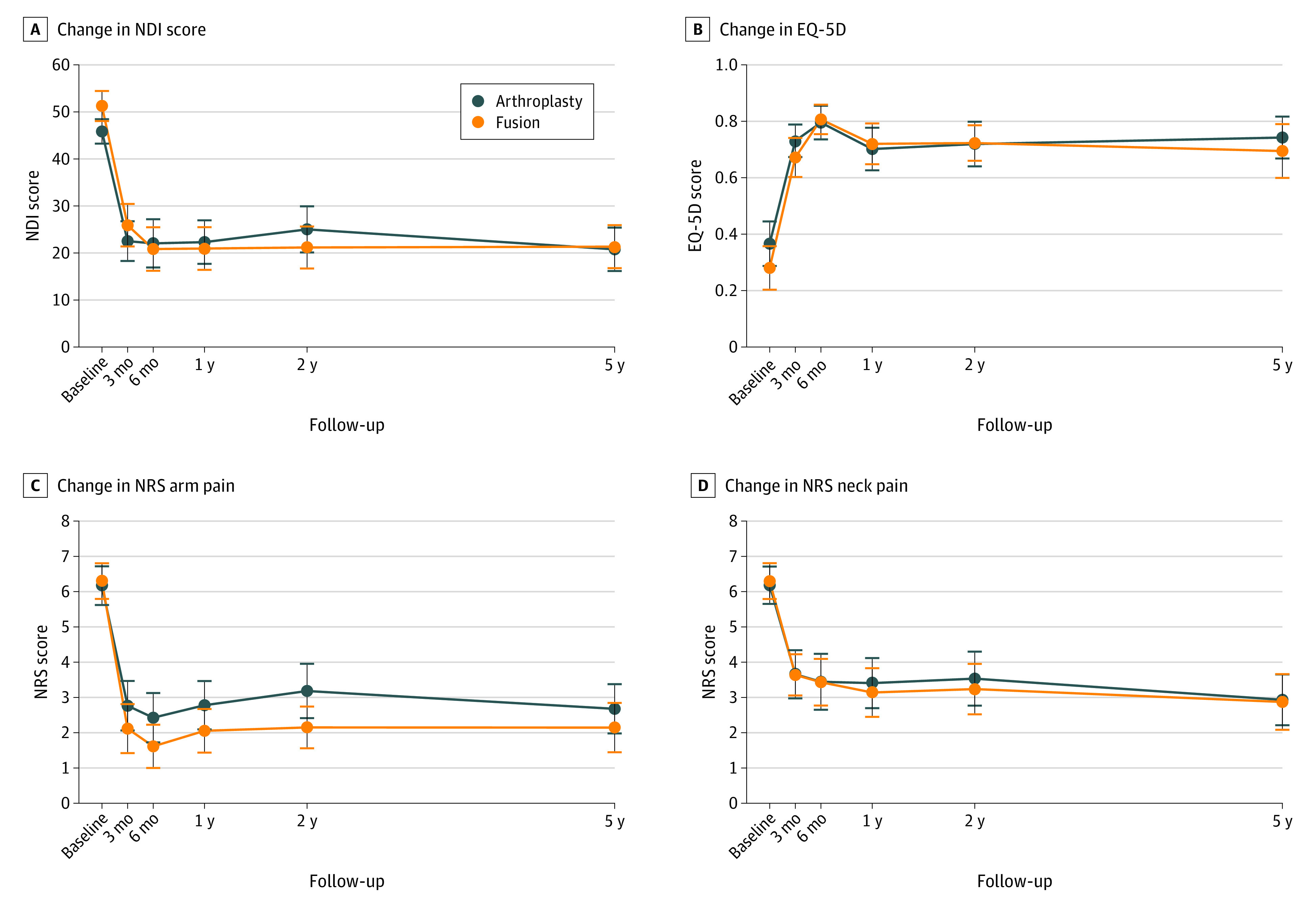
Changes in Patient-Reported Outcomes at 5-Year Follow-up EQ-5D indicates EuroQol-5D; NDI, Neck Disability Index; and NRS, numeric rating scales.

In the arthroplasty group, the mean NDI score decreased from 45.9 (95% CI, 43.3 to 48.5) points at baseline to 20.8 (95% CI, 16.2 to 25.4) points at 5 years (difference, 24.8 [95% CI, 19.8 to 29.9] points; *P* < .001). In the fusion group, the NDI score decreased from 51.2 (95% CI, 48.0 to 54.4) points at baseline to 21.3 (95% CI, 16.8 to 25.9) points at 5 years, (difference, 29.9 [95% CI, 24.0 to 35.9] points; *P* < .001). There was no significant difference in NDI score change between the 2 groups at 5 years (difference, 5.1 [95% CI, −2.6 to 12.7] points; *P* = .19) (Figure 2). As presented in Table 2, linear mixed models showed no significant difference between the groups at any time point during follow-up.

**Table 2.** Comparison of Outcome Measures Between Arthroplasty and Fusion Groups

Follow-up	Arthroplasty	Fusion	Mean difference	*P* value
**NDI score, mean (95% CI), points**^a^				
Baseline	45.9 (43.3 to 48.4)	51.3 (48.1 to 54.4)	NA	NA
3 mo	24.0 (19.9 to 28.1)	24.4 (20.1 to 28.6)	−0.4 (−6.3 to 5.6)	.90
6 mo	24.2 (20.0 to 28.4)	20.7 (16.2 to 25.2)	3.5 (−2.7 to 9.7)	.27
1 y	23.8 (19.7 to 28.0)	20.9 (16.6 to 25.2)	2.9 (−3.1 to 8.9)	.33
2 y	26.4 (22.2 to 30.5)	20.5 (16.3 to 24.7)	5.9 (−0.1 to 11.8)	.05
5 y	22.2 (18.0 to 26.3)	21.3 (17.0 to 25.6)	0.9 (−5.1 to 6.9)	.77
**EQ-5D-3L, mean (95% CI), points**^a^				
Baseline	0.37 (0.29 to 0.45)	0.28 (0.20 to 0.35)	NA	NA
3 mo	0.72 (0.66 to 0.79)	0.68 (0.62 to 0.74)	0.05 (−0.05 to 0.13)	.33
6 mo	0.79 (0.72 to 0.85)	0.81 (0.74 to 0.89)	−0.02 (−0.13 to 0.08)	.64
1 y	0.69 (0.63 to 0.76)	0.72 (0.66 to 0.79)	−0.03 (−0.12 to 0.06)	.53
2 y	0.71 (0.64 to 0.77)	0.73 (0.66 to 0.79)	−0.02 (−0.11 to 0.08)	.71
5 y	0.74 (0.68 to 0.81)	0.71 (0.63 to 0.79)	0.03 (−0.07 to 0.14)	.52
**NRS arm pain, median (range), points**^b^				
Baseline	6.0 (1.0 to 10.0)	6.5 (1.0 to 10.0)	NA	NA
3 mo	2.0 (0.0 to 8.0)	1.0 (0.0 to 10.0)	0.7 (−0.2 to 1.8)	.11
6 mo	2.0 (0.0 to 8.0)	1.0 (0.0 to 8.0)	1.1 (0.1 to 2.1)	.03
1 y	2.0 (0.0 to 8.0)	1.0 (0.0 to 7.0)	0.8 (−0.1 to 1.8)	.09
2 y	2.0 (0.0 to 10.0)	1.5 (0.0 to 8.0)	1.0 (0.0 to 1.9)	.04
5 y	2.0 (0.0 to 9.0)	2.0 (0.0 to 7.0)	0.6 (−0.3 to 1.6)	.20
**NRS neck pain, median (range), points**^b^				
Baseline	7.0 (0.0 to 10.0)	7.0 (1.0 to 10.0)	NA	NA
3 mo	3.5 (0.0 to 9.0)	3.0 (0.0 to 10.0)	0.1 (−0.8 to 1.0)	.83
6 mo	3.0 (0.0 to 9.0)	3.5 (0.0 to 8.0)	0.2 (−0.8 to 1.2)	.67
1 y	3.0 (0.0 to 9.0)	3.0 (0.0 to 9.0)	0.2 (−0.7 to 1.2)	.60
2 y	3.0 (0.0 to 10.0)	3.0 (0.0 to 10.0)	0.3 (−0.6 to 1.2)	.50
5 y	2.0 (0.0 to 8.0)	2.0 (0.0 to 10.0)	0.0 (−1.0 to 0.9)	.93

Abbreviations: EQ-5D-3L, EuroQol-5D; NA, not applicable; NDI, Neck Disability Index; NRS, numeric rating scales.

^a^ Baseline was reported as observed data, follow-up time estimated by linear mixed models.

^b^ Baseline was reported as observed data, and mean difference and *P *values were estimated by linear mixed models.

### Secondary Outcomes

There were no differences in changes of secondary outcome measures between the groups from baseline to 5 years (Figure 2). As shown in Table 2, there were no significant differences between the arthroplasty and fusion groups at 5 years in changes of arm pain (mean [SE] change, 3.5 [0.5] vs 3.1 [0.4]; mean difference, −0.4 [95% CI, −1.7 to 0.8] points; *P* = .47), neck pain (mean [SE] change, 3.0 [0.5] vs 3.4 [0.5]; mean difference, −0.5 [95 % CI, −1.7 to 0.8] points; *P* = .50), EQ-5D (mean [SE] change, 0.39 [0.04] vs 0.45 [0.06]; mean difference, −0.06 [95% CI, −0.22 to 0.10] points; *P* = .46), patients reoperated (10 patients [14.7%] vs 8 patients [11.8%]; *P* = .61), and adjacent segment disease (0 patients vs 1 patient [1.5%]; *P* = .32).

Overall, regardless of type of implant, there were significant improvements in all secondary outcome measures (Figure 2). EQ-5D index score improved from 0.37 (95% CI, 0.29 to 0.45) points at baseline to 0.74 (95% CI, 0.68 to 0.81) points at the 5-year follow-up for arthroplasty and from 0.28 (95% CI, 0.20 to 0.35) points at baseline to 0.71 (95% CI, 0.63 to 0.79) points at the 5-year follow-up for fusion. Likewise, median (interquartile range [IQR]) NRS arm pain improved from 6 (5-8) points at baseline to 2 (0-4) points at the 5-year follow-up for arthroplasty and from 6.5 (5-8) points at baseline to 2 (0-4) points at the 5-year follow-up for fusion, and median (IQR) NRS neck pain improved from 7 (5-8) points at baseline to 2 (0-6) points at the 5-year follow-up for arthroplasty and from 7 (5-8) points at baseline to 2 (0-5) points at the 5-year follow-up for fusion. For NRS arm pain, there was a significant difference in favor of fusion at 2 years, but the difference was not significant at 5 years (Table 2).

### Reoperations

There were 11 reoperations in 10 patients (14.7%) in the arthroplasty group and 9 reoperations in 8 patients (11.8%) in the fusion group (*P* = .61). All reoperations in the arthroplasty group were at the index segment. Four patients underwent reoperations owing to migration and anterior displacement of the prosthesis, and 2 of those underwent reoperation after more than 2 years. Seven reoperations were performed owing to persisting radiculopathy, 6 of whom underwent reoperation within 2 years, and 1 patient after 3 years. In the fusion group, 7 patients underwent reoperation after more than 2 years, all owing to radiculopathy. Only 1 patient underwent reoperation for what was considered clinical adjacent segment disease. The indications and time for reoperations are listed in Table 3.

**Table 3.** Indications and Timing for Reoperations

Patient			Time after surgery	Reason for reoperation	Segment
No.	Sex	Age, y, decade			
**Arthroplasty**					
1	Man	30s	8 mo	Persisting radiculopathy, removed arthroplasty device, inserted cage and plate	Index
2	Man	40s	10 mo	Persisting radiculopathy, operated with posterior foraminectomy	Index
3	Man	40s	10 mo	Persisting radiculopathy, removed arthroplasty device, inserted cage and plate	Index
4	Woman	40s	1 y, 2 mo	Persisting radiculopathy, removed arthroplasty device, inserted cage and plate	Index
5	Man	40s	1 y, 4 mo	Loosening, removed arthroplasty device, inserted cage and plate	Index
6	Woman	30s	1 y, 4 mo	Loosening, removed arthroplasty device, inserted cage and plate	Index
7	Man	40s	1 y, 5 mo	Persisting radiculopathy, operated with posterior foraminectomy	Index
8	Woman	50s	1 y, 8 mo	Recurrence of radiculopathy, removed arthroplasty device, inserted cage and plate	Index
9	Man	30s	2 y, 5 mo	Loosening, removed arthroplasty device, inserted cage and plate	Index
10	Woman	30s	3 y, 3 mo	Persisting radiculopathy, operated with posterior foraminectomy, second reoperation	Index
11	Man	40s	4 y, 3 mo	Loosening, removed arthroplasty device, inserted crista bone graft and plate	Index
**Fusion**					
1	Man	40s	3 wk	Had 2-segment spondylosis at inclusion (and hence should have been excluded), and was perceived to have a C7 radiculopathy at first, but was reevaluated after no effect of surgery, and reoperated at the adjacent segment, C5/C6	Adjacent
2	Woman	40s	1 y, 9 mo	Persisting radiculopathy, operated with posterior foraminectomy	Index
3	Woman	40s	2 y, 7 mo	Recurrence of radiculopathy, operated with posterior foraminectomy	Index
4	Man	40s	2 y, 8 mo	Developed radiculopathy 2 segments above primary surgery, operated with anterior discectomy and cage	2 Segments above index
5	Man	50s	2 y, 10 mo	Persisting radiculopathy, operated with posterior foraminectomy	Index
6	Woman	30s	3 y, 5 mo	Recurrence of radiculopathy, operated with posterior foraminectomy	Index
7	Man	40s	3 y, 9 mo	Recurrence of radiculopathy 2 segments above index, operated with posterior foraminectomy, second reoperation	2 Segments above index
8	Woman	40s	4 y, 6 mo	Developed radiculopathy at adjacent segment, operated with anterior discectomy and cage	Adjacent
9	Woman	30s	4 y, 6 mo	Developed radiculopathy 2 segments above primary surgery, operated with cage	2 Segments above index

### Blinding

The study participants were offered the opportunity to know which treatment they received at 2 years. At 5 years, 63 of 102 patients were still unaware of which device they received, including 28 of 54 patients (51.9%) in the arthroplasty group and 35 of 48 patients (72.9%) in the fusion group.

## Discussion

This randomized clinical trial found similar efficacy and reoperation rates for arthroplasty and fusion 5 years after surgical treatment for cervical radiculopathy. The improvements in all PROMs were sustained at 5 years for both interventions.

Our results are in accordance with a recent Swedish trial of artificial disc replacement vs fusion with 5 years of follow-up.^21,22^ However, our results are not consistent with several meta-analyses that show results in favor of arthroplasty.^11,23^ This may be owing to the blinding procedure used in our study reducing expectation bias. Blinding is important when there is some subjectivity in the assessment, ie, when PROMs are used. The fact that most of the patients were still unaware of which implant they received at 5 years suggests efficient blinding. Furthermore, the surgeons were blinded for the result of randomization until decompression of the nerve root was performed thus reducing the risk of performance bias during surgery.

There were no reoperations owing to implant subsidence in the arthroplasty group, as was reported in the Swedish trial.^21^ Four patients were reoperated owing to loosening of the arthroplasty device. However, most of the reoperations in this group were owing to persistent or recurrent radiculopathy. A 2020 meta-analysis by Badhiwala et al^24^ reported that for patients who underwent surgical treatment with fusion, the reoperation rate owing to adjacent segment disease was significantly higher at 5 years compared with patients treated with arthroplasty. Our findings are in dissonance with this meta-analysis, because we found only 1 patient underwent reoperation owing to clinical adjacent segment disease in the fusion group after 5 years. Well-defined indications for reoperations in arthroplasty studies are rare, and the reoperation rates vary in different studies. In case of severe pain at follow-up, surgeons’ opinions on either treatment may influence the decision to reoperate. In contrast to previous meta-analyses, we found that the reoperation rates were not significantly different between groups.^6,11^ As suggested in a 2020 meta-epidemiologic study by Kurian et al,^25^ this may be owing to publication bias, since earlier studies reported lower reoperation rates for arthroplasty than fusion.

Several manufacturers offer arthroplasty devices, and they have different biomechanical properties. Devices are considered constrained if they restrict motion in certain planes, semiconstrained if they allow physiological movement, and nonconstrained if they allow movement to an extent that range of motion is constricted by other structures in the spine, such as joints and soft tissue. This study used a nonconstrained device with a ball-and-socket design. The nonconstrained design might have a different impact on the adjacent segment compared with other designs, and this may have influenced the results. In the fusion group, a stand-alone polyetheretherketone cage implant was used. There was no anterior plating performed, which differs from most other studies. This could influence our results; however, a meta-analysis of studies on fusion by Cheung et al^26^ found no clinical benefit from the use of additional plating.

As arthroplasty and fusion have similar efficacy for cervical radiculopathy, we believe the choice of surgical strategy should rely on other factors, such as cost to the patient and health care system, length of surgical procedure, and surgeons’ preference. Operation time and cost clearly favored fusion in the NORCAT study.^12^ However, cost associated with the use of surgical implants may vary among different health care settings and reimbursement systems.

This study had several strengths. Some strengths of this study include high internal validity with randomized treatment allocation and an adequate sample size, long-term follow-up exceeding 80% of patients, blinding of surgeons until insertion of the implant, and successful blinding of patients.

### Limitations

This study has some limitations. The generalizability of this study is limited by the randomized design with highly selected patients. However, most patients treated for cervical radiculopathy are middle-aged adults who are otherwise relatively healthy and quite similar to our study population. We therefore consider our results generalizable to daily clinical practice.

Severe spondylosis at more than 1 segment was an exclusion criterion in our study. This was not based on systematic radiographic analysis prior to inclusion. Therefore, it is possible that some patients not meeting the criteria for arthroplasty may have been included in the study. This may favor the fusion group. Future studies should allow inclusion of patients with signs of degeneration in more than 1 cervical spine segment.

Additionally, we did not have well- or predefined criteria for performing reoperations. If patients reported persistent pain at follow-up, the surgeons’ opinion on either treatment may have influenced the decision whether to reoperate, ie, selection bias.

## Conclusions

This randomized clinical trial found similar efficacy and reoperation rates after arthroplasty vs fusion 5 years after surgical treatment for single-segment cervical radiculopathy. The large improvements in all PROMs after surgical treatment were sustained at 5 years for both interventions. We found no evidence for increased clinical adjacent segment disease in the fusion group. We conclude that both treatment modalities are equally good options for treating cervical radiculopathy.

## Notes

Supplement 1. Trial Protocol

Supplement 2. **eTable.** Complete List of Inclusion and Exclusion Criteria

Supplement 3. Data Sharing Statement
