# Supplementary material for: Effect of Arthroplasty vs Fusion for Patients With Cervical Radiculopathy: A Randomized Clinical Trial
Source: JAMA Netw Open. 2021 Aug 5;4(8):e2119606. doi: 10.1001/jamanetworkopen.2021.19606 (PMC8343489; doi:10.1001/jamanetworkopen.2021.19606)
Supplement: Supplement 1. — Trial Protocol [file jamanetwopen-e2119606-s001.pdf]

TREATMENT OF CERVICAL RADICULOPATHY WITH ARTHROPLASTY COMPARED WITH DISKECTOMI AND FUSION WITH CAGE (ACDF)

Clinical, radiological and biomechanical aspects

A randomized multicenter study

Øystein P Nygaard
Frode Kolstad
Bjarne Lied
Jarle Sundseth
Erling Myrseth
Jens Munch Ellingsen
Andreas Bliksås
Kjell Arne Kvistad
Gunnar Leivseth
Lars Gunnar Johnsen
Hege Andresen
John Anker Zwart

Trondheim

10.01.08

Authorized of the ethical committee 11.02.08

SUMMARY **2**

INTRODUCTION AND BACKGROUND **3**

AIM OF THE STUDY **4**

STUDY DESIGN **4**

PATIENTS (INCLUSION CRITERIA) **4**

EXCLUSION CRITERIA **4**

INVESTIGATIONS **5**

TREATMENT **5**

STATISTICAL METHOD **5**

OUTCOME VARIABLES **6**

FOLLOW UP **6**

ETHICS **7**

SECURITY **7**

REPORT **7**

PATIENT INFORMATION AND INFORMED CONSENT **7**

TIME PLAN **7**

APPLICATION ABOUT APPROVAL **7**

FUNDING **8**

SUB-PROJECTS **8**

- 2) MRI STUDIES 10
- 3) DCRA STUDIES 11
- LITERATURE 13

LITERATURE **13**

ATTACHEMENTS **13**

Summary

Cervical radiculopathy due to disc herniation or spondylosis is a painful condition which often can be treated symptomatic in pending of spontaneous improvement.

However, some patients need operative treatment owing to persistent pain.

The objective of surgery is to relieve the nerve root which is compromised, and at the same time, stabilize the segment with fusion (ACDF).

The clinical results of this procedure are documented in several prospective studies, where approximately 70 % of the patients report improvement or considerable improvement post-operatively. The long-term results are somewhat poorer due to new symptoms from the level operated upon or from the disc above or below the fused segment. Several studies conclude that symptoms from so called “adjacent level disease” develop in roughly 25 % of patients within the first 10 years subsequent to fusion surgery. However, most of these studies have methodological weaknesses.

Whether fusion surgery leads to increased load above or below the operated level, with resulting increased spondylosis, or this is the natural development of spondylosis, is still not clear.

In the last years, a new alternative to fusion surgery has developed. Cervical arthroplasty (artificial discs) was introduced around 1990 and is supposed to replace the symptomatic disc with preservation of movement after decompressive surgery for the compromised nerve root. There are several minor randomized studies where cervical arthroplasty is compared with discectomy and fusion, but there is only one recently published study that found any clinical benefit in favour of arthroplasty. The main argument for using these artificial discs is preservation of segment motion, and hopefully reducing the risk of developing “adjacent level disease”. This is, however, not confirmed in previous studies.

The aim of the study is to compare the artificial discs against the traditional method with fusion surgery. The study is a prospective, randomized, single blinded, multicenter study where all Norwegian hospital departments who perform surgery on cervical disc disease attend.

Patients with single level disease (disc herniation or spondylosis) with compression of one nerve root are suitable for inclusion.

Patients with myelopathy are not included.

The study focuses on three areas:

1) The clinical effect of the two different interventions estimated with established outcome measures.
2) A health economical evaluation to compare cost versus clinical effect of the two surgical methods
3) A biomechanical and radiological (MRI) mapping to evaluate if the progress of spondylosis above or below the operated level differ among the two interventions

Power estimates have indicated that the study needs a total of 146 patients. External validity will be evaluated by systematic registration of all patients considered as candidates for inclusion.

The patients will be controlled at regular intervals for a minimum of two years.

Initialisation is planned in June 2008 and the inclusion period is estimated to two years.

The study is coordinated from The National centre for spinal disorders at St. Olavs Hospital in Trondheim.

Introduction and background

Cervical radiculopathy on the basis of disc herniation or spondylosis is a painful condition which most often can be treated symptomatically in pending of spontaneous improvement. However, some patients have persistent pain demanding operative treatment. The clinical condition is characterized by neck pain, radiating to one or both of the upper extremities. Most often the patient has associated neurological symptoms like numbness, paresthesias (tingling sensations) and paresis.

Approximately 500 patients annually are operated on due to cervical radiculopathia each year in Norway. In USA more than 100 000 are treated surgically. The standard operative treatment was described roughly 50 years ago and consists of anterior decompression and fusion. The herniated or degenerated disc is removed, the nerve root decompressed and the level of disc disease fused. The fusion is performed with implantation of a "cage" which is inserted in the decompressed disc space. The cage is made of a synthetical material (Poly-Ether-Ether-Ketone, PEEK). The object with the operative treatment is to relieve the compressed nerve root and perform a fusion of the segment. The clinical results are well documented in prospective studies where approximately 70% report improvement or considerable improvement after surgery. Several studies conclude that symptoms arising from disc above or below the level operated upon, so called "adjacent level disease" develop in close to 25% of the patients during the first 10 years after discectomy and fusion. Most of these studies have methodological weakness. Whether fusion surgery cause increased load on neighbour disc levels with new symptoms as a consequence or if this is a natural development of spondylosis, is still unclarified.

In the last years, a new alternative to fusion surgery has developed. Cervical arthroplasty (artificial discs) was introduced around 1990 and is supposed to replace the symptomatic disc with preservation of movement after decompressive surgery for the compromised nerve root. There are several minor randomized studies where cervical arthroplasty is compared with discectomy and fusion, but there is only one recently published study that confirms any clinical benefits in favour of artificial disc implant. Nevertheless, arthroplasty is more and more becoming the new standard treatment of cervical radiculopathy. The main argument for using these artificial discs is preservation of segment motion and hopefully reducing the risk of

developing “adjacent level disease”. The scientific documentation on this aspect is, however, uncertain. The operative technique with artificial discs is somewhat more demanding than the procedure using a cage. In addition, arthroplasty is probably more expensive.
The attitude towards this new technology among Norwegian neck-surgeons has so far been expectant. There is a consensus that cervical arthroplasty is to be used exclusively in a prospective randomized study.
A systematic registration of the biomechanical conditions and radiological (MRI) findings in adjacent levels over several years is the only chance to clarify whether artificial discs represent a better surgical alternative for the patients than cage implant and fusion.
A health economic evaluation to illustrate cost versus clinical effect (ICER-incremental cost effect ratio) is also essential in order to be able to evaluate the benefit of this new technology.

Aim of the study

The hypothesis is that anterior discectomy and implant of an artificial disc gives equally good clinical result compared with anterior diskectomi and fusion with cage, and at the same cost. There is no difference in development of adjacent level disease when comparing the two surgical methods.
0-hypothesis: There is no difference between the two interventions when using primary and secondary outcome variables.

Study design

Prospective, randomized, single-blinded, multicenter study

Patients (inclusion criteria)

Age 25 - 60 years. Patients with clinical C6 or C7 root radiculopathy with corresponding radiological findings with or without neurological symptoms. Mechanical provoked pain which aggravate with physical activity or positive Spurlings test. Radiological nerve root compression on the basis of disc herniation or spondylosis. NDI > 30 points
The patient has not responded to non-operative treatment and has no sign of improvement during the last 6 weeks.

Exclusion criteria

Significant spondylosis involving more than one level
Intramedullary changes on MRI
Ankylosis in adjacent level
Clinical suspicion of myelopathy
Chronic generalized pain syndrome
Infection

Active cancer disease
Rheumatoid arthritis involving the cervical spine
Previous trauma involving the cervical spine
Pregnancy
Allergy against contents in cage/artificial disc
Previous neck surgery
Psychic or somatic illness that causes the patient not to be suitable for the study
The patient does not understand Norwegian orally or in writing.
Abuse of medication/narcotics

Investigations

A) Prior to inclusion: MRI, not older than 4 months
B) After inclusion: x-ray of the cervical column with flexion and extension images
   MRI with flexion and extension images will be performed at the hospitals where this technique is available.
C) After surgery: X-ray of the cervical column with flexion and extension images on day one and at 8-12 weeks follow up. MRI and x-ray with flexion and extension images 8-12 weeks, 1 year, 2 and 5 years post.op.

Treatment

The surgical treatment has two arms:
After decompression of the nerve root through an anterior approach, the patient will be randomized to either implantation of an artificial disc or a cage. (PEEK)

Statistical method

The difference between the two groups will be presented as mean (95% CI or Odds ratio for the categories). Base line data is compared. Differences within the groups from baseline to follow up will be compared with paired (two sided) t-test.
Multiple regression analysis will be used to perform predictor analysis. Randomizing is performed through a web based access to a special program at the department for applied research at NTNU (University of Trondheim).
Randomizing come to pass during operation when decompression is completed and before insertion of the implant.
The patient will be blinded for which type of implant he or she has received. This is done in order to reduce anticipation-bias with both the patient and the therapists.

Power estimate:

Primary outcome variable is NDI (0-100%). SD (Standard Deviation) in previous studies concerning cervical disc herniation is approximately 18%. Clinical relevant change is placed at 10%. Significance level 5%, power 80%. This gives a total of 104 patients needed for the study. Correction for the multicenter design and lost to follow up at 30% gives a total of 146 patients attending the study.

There will be performed analysis of all patients included ("intention to treat"), and all patients who drop out of the study for some reason will be accounted for.

All patients who are considered for inclusion will be registered and the reason for not attending recorded (external validity).

Outcome variables

Main outcome variable:

Neck Disability Index (NDI)

Secondary outcome variables:

EQ5-D

SF-36

Numeric pain in neck and arm

Symptoms of dysphagia using "dysphagia score"

Complications, rate of reoperations, patients satisfaction.

Co-morbidity will be registrated

The data is collected and installed in a database at the National centre for spinal disorders at St.Olavs Hospital, Trondheim.

All departments will administer a research secretary and a doctor who will be accountable for inclusion and operative treatment.

Follow up

Follow up: 1 day after surgery, 8-12 weeks, 6 months, 1 year, 2 years and 5 years.

The outcome variable will be registered at every follow up. Clinical controls after 8-12 weeks, 1 year and 2 years.

	Clinical control	Questionnaires	Health economy	MRI	X-ray with flex/ex images
1.day post.opr					✓
8 - 12 weeks	✓	✓	✓	✓	✓
6 months		✓	✓		
1 year	✓	✓	✓	✓	✓
2 year	✓	✓	✓	✓	✓
5 year		✓		✓	✓

Ethics

It is most important to select the proper patients, where there is agreement regarding honest uncertainty about which operative method being most suitable.
If problems arise during arthroplasty, cage implant will be performed.

Security

All complications will be registered, and all serious adverse affects will be reported to the study committee. In case of severe complications, the ethical committee will be notified and the study considered stopped.
The rate of complications in both surgical techniques is well known and do not differ in the literature.

Report

An interim analysis will be performed when 50% of the patients are included.

Patient information and informed consent

Please see attachment.

Time plan

The protocol is composed during autumn 2007. The regional ethical committee will receive the application in jan.2008. The study is planned initialised in June 2008. Inclusion period is 2 years. The first article is expected published in 2010. The study is concluded in 2018.

Application about approval

Approved by The Regional Committee for Medical and Health Research, Midt-Norge, Norwegian Social Science Data Services (NSD) and The Data Inspectorate.

Sub-projects

1) Health economy

A health economic evaluation of the two interventions is necessary in order to be able to analyse whether the cost connected to cervical disc arthroplasty can be justified, when taking enhanced health profit into account. We plan a complete survey of costs both during the hospitalization and after the patient has been discharged.

Cost analysis:
In this analysis we will evaluate if there are significant differences regarding the costs comparing the two different surgical techniques. While in the ”cost-utility” analysis mean costs are used, in this sub-study the estimates regarding different cost components attached to the hospital management, follow up, and rehabilitation will be evaluated and compared among the different participating hospitals. Consequently, identification of the most expensive segments in the chain of treatment will be possible, which is important in order to find the most efficient organisation of treatment. The analyses will be completed utilising two different angels: One analysis where the hospital costs is included, and one which include the expenses involving other parts of the health sector, the patients, relatives, as for the rest of society (table 1). If the analyses indicate that one method of treatment is more costly than the other and health profits among the two turn out to be equal, the method with lowest costs is to be preferred.

Table 1: Collection of information about costs

Group of costs	Variable	Source
Application of hospital resources	Minute rate on operation, anaesthesia and recovery. 24 hour rate on department stay	Minute rate is determined in advance through review of costs associated with the surgical department.<br>Executed in cooperation with SINTEF (CPP =Cost Per Patient). Information concerning time use is registered on the surgical anaesthesia forms and in the patient’s journal.
Use of health services outside the hospital	Expenses connected to physical therapy, general practitioner, and other therapists	Health diary, the patient complete/fill in running costs during the period from 0-3 months, 3-6 months, 6-12 months and 12-24 months
Patients costs and possibly relatives outlays	Travel to and fro therapists , pain relievers, use of medical and paramedical services	Health diary, the patient complete running costs during the period from 0-3 months, 3-6 months, 6-12 months and 12-24 months
Society costs	Medical certificate, disablement, transport expenses, remedies	Social security office. The patient has given informed consent about gathering information.

By using the health diary, it will be possible to look at variations regarding the patient's use of health services outside the hospital. If arthroplasty involve less need for rehabilitation, we will be able to measure this by looking at the patients reduced need for physical therapy and fewer days of incapacity. In addition, it is possible that the strain on relatives will be less. Cost variations can be explained according to more than just method of treatment and will therefore be included in the analyses.
Standard regression analyses will be used to analyse differences in costs according to which surgical technique being performed. One will also evaluate the possible benefit of using multiple level-analyses to look at variations at hospital level.

In this study the treatment of patients is equally organized which means admittance in the hospital the day prior to planned surgery and discharged the first or second day after surgery. If the study indicates that one of the surgical techniques is associated with shorter hospital stay, the question about organizing emerges. Therefore, sensitivity analyses will be performed, which means analysing total expenses by changing single elements in the chain of treatment.

Cost-utility analysis:
In this analysis a full health economic evaluation is performed to estimate health effect versus costs according to which method of surgery being selected. This is important in the purpose of being able to choose the best operative method for this group of patients. The basis of the analysis is standard methods within health economic evaluation. To accomplish this, we will measure the improvement in state of health after surgery by quality adjusted years of one's life (QUALY`s), which is a standard measure in cost-utility analyses. QUALY is a measure of health effect which includes both duration and impact on quality of life. Quality of life is measured with a scale from 0 to 1, where 0 refers to death and 1 to perfect health. The variable quantity of this measure reflects the society's preferences associated with health, and might influence the priority setting of treatment options towards certain patient groups. Quality of life is measured using validated questionnaires. EQ-5D is the most widely used instrument in Europe, while the generic questionnaire SF-36 includes more proportions and is widely used in evaluation of quality of life regardless of disease. Both can be used to estimate QUALY. The quality of life forms is to be completed before operation and at 6-8 weeks, 6 months, 1, 2 and 5 years. Change in index over this time period forms the basis for calculating effect on quality adjusted years of one life (QUALY).
Collection of information concerning costs will take place as described in line table 1.
With basis in a tree of resolution, it is possible to estimate expected costs and QUALY`s comparing the two different surgical techniques. Based on the analysis, we gather information about:

- Cost per QUALY for both interventions
- Effect on cost per QUALY by including costs outside the hospital
- Incremental cost-effectiveness ratio (ICER), which means change in cost by change in effect (QUALY). For instance, if disc arthroplasty is more expensive than cage, but the effect using QUALY`s is greater, ICER will then be an expression for the expenses involved when changing from cage to artificial disc (see figure 1 for more possible outcome).

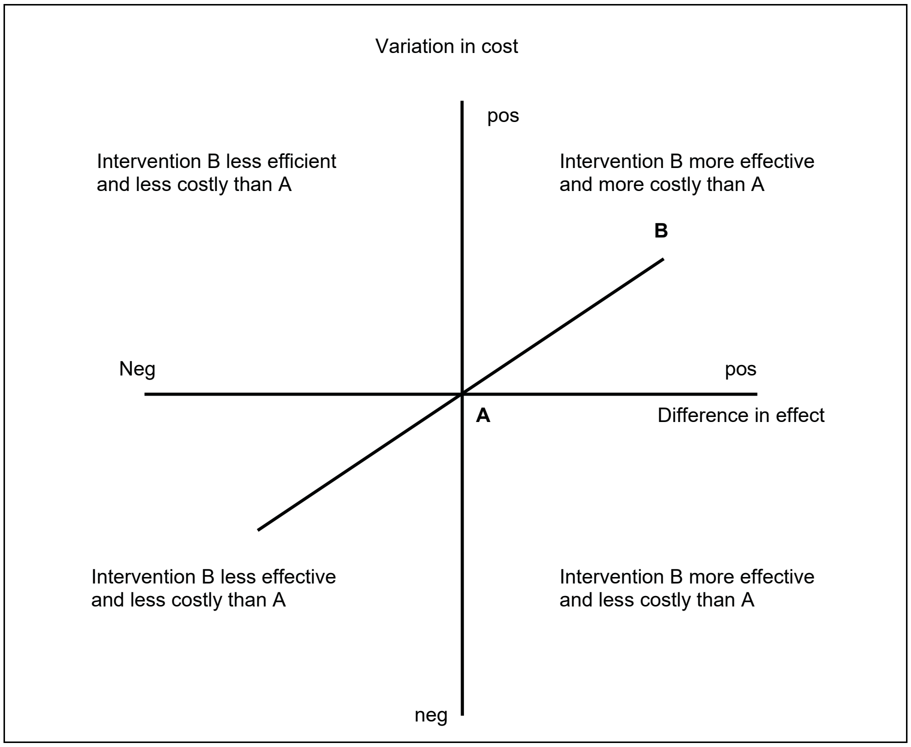

Figure 1: illustrate incremental cost-effectiveness ratio

2) MRI studies

Assessment of MRI changes in level of symptomatology and neighbouring level regarding "adjacent level disease". MRI will be performed after 8 – 12 weeks, 1 year, 2 and 5 years to evaluate changes in level above and below the operated disc.
MRI myelography and functional MRI will be performed where this technology is available, to evaluate if these methods have predictive qualities considering effect of treatment.

<u>Planned sequences:</u>

1. Sag TSE T2, 3mm slice.
2. Sag T1, 3mm slice
3. Tra TSE T2, 3mm slice

4. 2 oblique sag, 3mm slice

- Transverse slices are placed in the right level and angled parallel with the operated disc.
- Transverse TSE is used to reduce artefacts from the artificial disc. Applicate high turbo factor (ex 20) and low band width.
- The objective with oblique sag series is to portray the root canals in the way we are used to look at them on plain oblique x-ray images of the cervical spine.
- Put cor and tra localize slices perpendicular on a sagittal series. This eases the later centering.
- The oblique sagital slices is placed perpendicular on the facet joints. Use the transversal slices on the localizer.

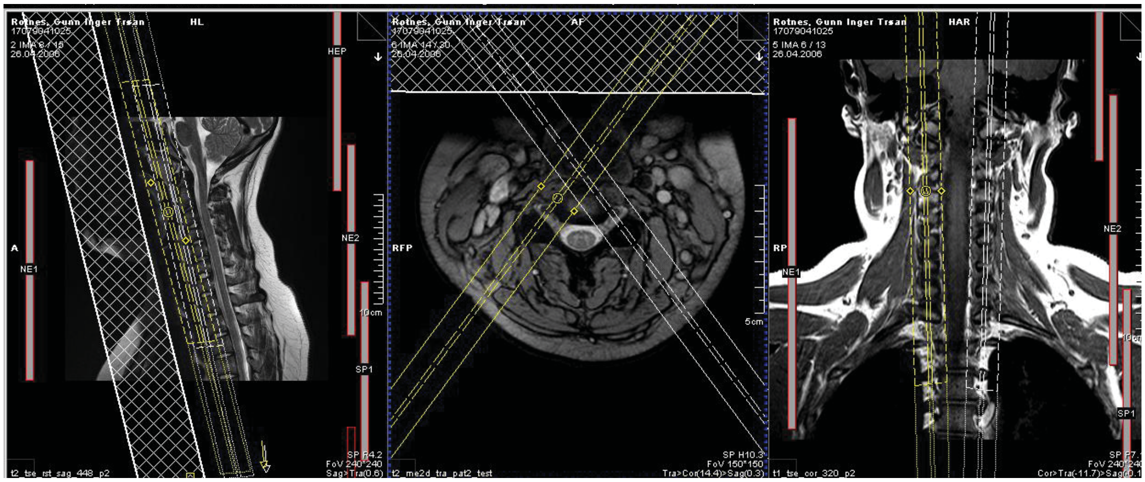

3) DCRA studies

There will be performed functional x-ray images with flexion/extension views of the cervical spine on all patients before surgery, the first day after surgery and at 8 - 12- weeks,1, 2 and 5 years, making DCRA analyses possible. DCRA (Distortion Compensated Roentgen Analysis) is a well known method established at NTNU and is used in several papers during the last 5 years. This method makes it possible to evaluate the function of the artificial disc regarding translation and angulation. It is also usable to evaluate if a spondylodesis using a cage is successful. In addition, data about change of motion in neighbouring levels over time is received. This is highly useful regarding evaluating the risk of developing “adjacent level disease”. When the pictures are taken, it is important to include C2 and C7.

Measurement protocol

To perform DCRA, the contours of the vertebrae are mapped and digitized. Series of computer programs check geometric properties of the contours, objectively locate vertebral ‘corners’ and calculate the parameters.

Measurements of rotational and translational segmental motion as well as of disc height and dorsoventral displacement are performed by DCRA. This method compensates for distortion caused by axial rotation, lateral tilt and off-centre positioning of the spine. This permits to process radiographs taken in normal clinical settings. Knowledge of the exposure geometry is not required. All motion segments imaged on a lateral radiograph can be evaluated.

Sagittal plane rotational motion is obtained in degrees. Dorsoventral displacement and translational motion, disc height and vertebral height are determined in relative units, i.e. divided by the individual, mean vertebral depth. This is done in order to compensate for variations in radiographic magnification and stature. Possible subsidence of the prosthesis will be measured as well.

Data collection and analysis

Sagittal plane motion of the segments C0/C1 to C6/C7 is determined from the pre- and postoperative pairs of flexion-extension radiographs. In the operated segment, disc height (or postoperatively: height of the intervertebrale space) and dorsoventral displacement will be determined from the pre- and postoperative radiographs taken in extension. As disc height and displacement (as defined here) both depend on the angle of lordosis, and in order to permit comparison with normal data, disc height and displacement are corrected to standard angles of lordosis.

Rotational and translational motion as well as disc height and dorsoventral displacement are compared with previously determined normal data. As the magnitude of translational motion depends linearly on the magnitude of rotational motion, actual translational motion is also compared with that motion predicted for a normal subject under the individual magnitude of rotational motion. Thus, the comparison between actual and predicted translational motion is independent of the magnitude of rotational motion performed by the patient. For the segments instrumented with arthroplasty, the deviation of disc height and displacement from the norm is expressed in standard deviations S of the appertaining distribution in the normal population. This permits to pool disc height and displacement data from all patients studied.

Literature

1. Sasso RC, Smucker JD et al. Artificial Disc Versus Fusion, Spine 2007; Vol 32, No 26: 2933-2940.

2. Kolstad F, Nygaard OP, Leivseth G. Segmental Motion Adjacent to Anterior Cervical Arthrodesis. SPINE 2007 Vol 32, No 5: 512-517

3. Kolstad F, Myhr G, Kvistad KA, Nygaard OP, Leivseth G. Degeneration and height of cervical discs classified from MRI compared with precise height measurements from radiographs, European Journal of Radiology 2005; 55, 415-420.

4. Praveen V. Mummaneni, Burkus JK, Haid RW et.al. Clinical and radiographic analysis of cervical disc arthroplasty compared with allograft fusion: a randomized controlled clinical trial. J Neuroseurg Spine 2007; 6: 198-209

5. Pimenta L, McAfee PC, Cappuccino A et.al. Superiority of Multilevel Cervical Arthroplasty Outcomes Versus Single-Level Outcomes, Spine 2007; Vol 32 No 12: 1337-1344.

6. Chang UK, Kim DH , Lee MC et.al, Range of motion change after cervical arthroplasty with ProDisc-C and Prestige artificial discs compared with anterior cervical discectomy and fusion, J Neurosurg Spine 2007; 7: 40-46

7. Ishihara H, Kanamori M, Kawaguchi Y et.al. Adjacent segment disease after anterior cervical interbody fusion, The Spine Journal 2004; 4: 624-628

8. Robertson JT, Papadopoulos SM et.al. Assessment of adjacent-segment disease in patients treated with cervical fusion or arthroplasty: a prospective 2-year study. J Neurosurg Spine 2005; 3: 417-423

9. Nabhan A, Ahlhelm F, Pitzen T et.al. Disc replacement using Pro-Disc C versus fusion: a prospective randomised and controlled radiographic and clinical study. Eur Spine J 2007; 16: 423-430

10. Phillips FM, Garfin SR. Cervical Disc Replacement, Spine2005; Vol 30, No175: 527-533

11. Lied B, Sundseth J, Helseth E, Immediate (0-6), early (6-72) and late (72---) complications after anterior cervical discectomy with fusion for cervical disc degeneration. Acta Neurochirurgica (Wien) 2007; DOI 10.1007/s00701-007-1472-y

12. Yoon DH, Yi S, Shin HC et.al. Clinical and radiological results following cervical arthroplasty. Acta Neurochirurgica (Wien) 2006; 148: 943-950

13. Sekhon LHS, Duggal N, Lynch JJ et.al. Magnetic Resonance Imaging Clarity of Bryan, Prodisc-C, Prestige LP, and PCM Cervical Arthroplasty Devices. Sine 2007; Vol 32, No 6: 673-680

Attachments

Informed consent
