# Supplementary material for: Effect of Arthroplasty vs Fusion for Patients With Cervical Radiculopathy: A Randomized Clinical Trial
Source: JAMA Netw Open. 2021 Aug 5;4(8):e2119606. doi: 10.1001/jamanetworkopen.2021.19606 (PMC8343489; doi:10.1001/jamanetworkopen.2021.19606)
Supplement: Supplement 2. — eTable. Complete List of Inclusion and Exclusion Criteria [file jamanetwopen-e2119606-s002.pdf]

Supplemental Online Content

Johansen TO, Sundseth J, Fredriksli OA, et al. Effect of arthroplasty vs fusion for patients with cervical radiculopathy: a randomized clinical trial. *JAMA Netw Open*. 2021;4(8):e2119606. doi:10.1001/jamanetworkopen.2021.19606

eTable. Complete List of Inclusion and Exclusion Criteria

This supplemental material has been provided by the authors to give readers additional information about their work.

© 2021 Johansen TO et al. *JAMA Network Open.*

eTable. Complete List of Inclusion and Exclusion Criteria

Inclusion criteria	Age between 25 and 60 years<br>C6 or C7 radiculopathy with corresponding MRI finding<br>Mechanically provoked pain which aggravates with physical activity or positive Spurling's test<br>NDI≥30%<br>No response to non-operative treatment<br>No clinical improvement during the last 6 weeks prior to surgery
Exclusion criteria	Significant spondylosis involving more than 1 level<br>Adjacent level ankylosis<br>Intramedullary changes on MRI<br>Clinical suspicion of myelopathy<br>Chronic generalized pain syndrome<br>Infection or active cancer<br>Rheumatoid arthritis involving cervical spine<br>Previous trauma involving the cervical spine<br>Pregnancy<br>Allergy towards contents in cage/arthroplasty device<br>Previous neck surgery<br>Mental or somatic injury which renders the patient unsuitable for the study<br>The patient does not understand Norwegian written or orally<br>Abuse of medication/narcotics

© 2021 Johansen TO et al. *JAMA Network Open.*
