# Supplementary material for: Effect of Arthroplasty vs Fusion for Patients With Cervical Radiculopathy: A Randomized Clinical Trial
Source: JAMA Netw Open. 2021 Aug 5;4(8):e2119606. doi: 10.1001/jamanetworkopen.2021.19606 (PMC8343489; doi:10.1001/jamanetworkopen.2021.19606)
Supplement: Supplement 3. — Data Sharing Statement [file jamanetwopen-e2119606-s003.pdf]

Data Sharing Statement

Johansen. Effect of Arthroplasty vs Fusion for Patients With Cervical Radiculopathy. *JAMA Netw Open*. Published August 05, 2021. doi:10.1001/jamanetworkopen.2021.19606

Data
Data available: No
